# Transcatheter non-acute retrieval of the tine-based leadless ventricular pacemaker

**DOI:** 10.1093/europace/euae256

**Published:** 2024-10-07

**Authors:** Moritoshi Funasako, Pavel Hála, Marek Janotka, Jan Šorf, Lucie Machová, Jan Petrů, Milan Chovanec, Jan Škoda, Lucie Šedivá, Jaroslav Šimon, Libor Dujka, Vivek Y Reddy, Petr Neužil

**Affiliations:** Cardiology Department, Na Homolce Hospital, Roentgenova 37/2, 15030 Prague, Czech Republic; Cardiology Department, Na Homolce Hospital, Roentgenova 37/2, 15030 Prague, Czech Republic; Cardiology Department, Na Homolce Hospital, Roentgenova 37/2, 15030 Prague, Czech Republic; Medtronic, Minneapolis, MN, USA; Cardiology Department, Na Homolce Hospital, Roentgenova 37/2, 15030 Prague, Czech Republic; Cardiology Department, Na Homolce Hospital, Roentgenova 37/2, 15030 Prague, Czech Republic; Cardiology Department, Na Homolce Hospital, Roentgenova 37/2, 15030 Prague, Czech Republic; Cardiology Department, Na Homolce Hospital, Roentgenova 37/2, 15030 Prague, Czech Republic; Cardiology Department, Na Homolce Hospital, Roentgenova 37/2, 15030 Prague, Czech Republic; Cardiology Department, Na Homolce Hospital, Roentgenova 37/2, 15030 Prague, Czech Republic; Cardiology Department, Na Homolce Hospital, Roentgenova 37/2, 15030 Prague, Czech Republic; Cardiology Department, Na Homolce Hospital, Roentgenova 37/2, 15030 Prague, Czech Republic; Department of Cardiology, Icahn School of Medicine at Mount Sinai, New York, NY, USA; Cardiology Department, Na Homolce Hospital, Roentgenova 37/2, 15030 Prague, Czech Republic

**Keywords:** Leadless cardiac pacemaker, Micra transcatheter pacing system, Leadless pacemaker retrieval, Leadless pacemaker reimplantation

## Abstract

**Aims:**

We report our single-centre experience of mid-term to long-term retrieval and reimplantation of a tine-based leadless pacemaker [Micra transcatheter pacing system (TPS)]. The TPS is a clinically effective alternative to transvenous single-chamber ventricular pacemakers. Whereas it is currently recommended to abandon the TPS at the end of device life, catheter-based retrieval may be favourable in specific scenarios.

**Methods and results:**

We report on nine consecutive patients with the implanted TPS who subsequently underwent transcatheter retrieval attempts. The retrieval system consists of the original TPS delivery catheter and an off-the-shelf single-loop 7 mm snare. The procedure was guided by fluoroscopy and intracardiac echocardiography. After an implantation duration of 3.1 ± 2.8 years (range 0.4–9.0), the overall retrieval success rate was 88.9% (8 of 9 patients). The mean procedure time was 89 ± 16 min, and the fluoroscopy time was 18.0 ± 6.6 min. No procedure-related adverse device events occurred. In the one unsuccessful retrieval, intracardiac echocardiography revealed that the TPS was partially embedded in the ventricular tissue surrounding the leadless pacemaker body in the right ventricle. After retrieval, three patients were reimplanted with a new TPS device. All implantations were successful without complications.

**Conclusion:**

A series of transvenous late retrievals of implanted TPS devices demonstrated safety and feasibility, followed by elective replacement with a new leadless pacing device or conventional transvenous pacing system. This provides a viable end-of-life management alternative to simple abandonment of this leadless pacemaker.

## Graphical Abstract

**Graphical Abstract:**
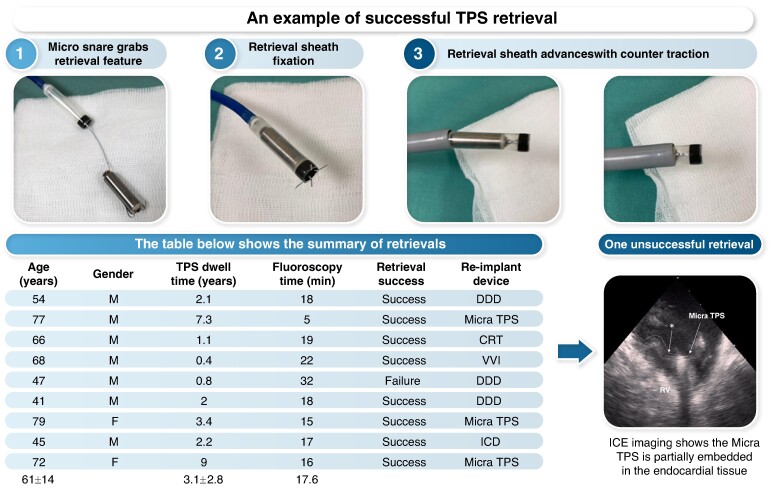

**What’s new?**

- All the attempted nine retrievals were performed without any complications. One retrieval failed because the Micra capsule was partially embedded in the surrounding endocardial tissue in the right ventricle.
- Immediate implantations of new Micra transcatheter pacing system (TPS) after retrievals were successfully performed in all three attempted cases without any abnormal electrical parameters.
- Five out of nine patients underwent device upgrade to dual-chamber pacemaker, implantable cardioverter defibrillator, or cardiac resynchronization therapy after the TPS retrieval trial. Device upgrades can be managed relatively easily among patients with leadless pacemakers.

## Introduction

The cardiac pacing system has a unique history in the past decades, and the improvement of the technology has contributed to reducing complications.^1^ Leadless cardiac pacemakers (LPs) are safe and effective alternatives to conventional transvenous pacemakers for patients who require single-chamber ventricular pacing: LPs avoid pocket-related and lead-related complications while achieving as long battery lives as conventional pacemakers.^2–4^ The transcatheter pacing system (TPS; Micra; Medtronic, Minneapolis, MN, USA), which is currently one of four commercially available LPs for clinical practice in EU countries, exhibited a high acute implantation success rate and sustainable pacing performance in the global prospective studies.^5^ Albeit these promising results, the lifetime management of these TPS devices seems to be a future concern as the manufacturer recommends abandoning the implanted devices at its end of life. Unlike traditional devices that are susceptible to infection involving the transvenous leads and device pockets, potential infectious complications associated with the TPS LP are quite infrequent.^6–8^ In certain conditions, retrievability of long-lasting implanted leadless pacemakers may be beneficial. Indeed, another LP with a longer, thinner body and a helix fixation that is designed to screw into the RV endocardial tissue comes with a dedicated transcatheter retrieval kit provided by the manufacturer. The successful retrieval rate of this helix-fixation LP has been reported to be around 80–95%. We recently reported a successful retrieval of a 9-year-old LP.^9–11^ However, this LP was withdrawn from the market due to preliminary battery depletion and is currently unavailable commercially. Although several successful acute and intermediate-term TPS retrieval cases have been reported,^11–13^ the feasibility and safety of late retrieval of TPS LPs are unclear. Therefore, we report our single-centre transvenous retrieval experience mid-term to long-term after TPS implantation.

## Methods

### Study participants

The present study included nine patients who required single-chamber ventricular pacemaker pacing due to symptomatic bradycardia, who underwent implantation of the TPS in the right ventricle (RV) in our institution between September 2014 and January 2019. The detailed TPS implantation technique has been previously described.^3^ All patients were well informed before the initial implantation procedure of the future possibility of device retrieval or abandonment—depending on the necessity, risks, and advantages of each choice. In this series, we included consecutive patients who met reasonable indications for the Micra retrieval and agreed with the transvenous retrieval procedure. Written informed consent was obtained from each patient before the retrieval procedure. The study was approved by the Institutional Ethics Committee of Na Homolce Hospital and was conducted under the Declaration of Helsinki.

### Definition of procedure success and complications

Successful TPS device retrieval was defined as the complete removal of the LP including nitinol tines without any missing parts.^9^ Any serious adverse events within 30 days after the device retrieval were meticulously recorded. The study applies a standard definition of peri-procedural serious adverse events: any device- or procedure-related untoward medical occurrence leading to death or a deterioration in the patient’s health that resulted in life-threatening illness or injury, permanent impairment of a body structure or a body function, and prolonged inpatient of prolonged hospitalization or medical or surgical intervention to prevent life-threatening illness, injury, or permanent impairment to a body structure or a body function.^10^

### Retrieval procedure

The TPS device is designed for acute retrieval in the event of repositioning before releasing the device: The proximal portion of the TPS contains a retrieval feature knob that enables operators to engage the proximal part of the device with a snare, as there are no specifically designed retrieval tools available for this purpose. Two experienced operators performed all retrieval procedures under conscious sedation. The cardiac surgical department and cardiac anaesthesiology department supported the case as a backup team in the event of severe cardiac tamponade, valve injury, or any complications that require emergency surgical operation. The retrieval catheter system was the same system that was originally designed for TPS implantation. After the 23-French (ID) sheath for TPS insertion was introduced via the right femoral vein, the contrast was injected in the RV through a pigtail catheter to delineate the TPS position. An intracardiac echocardiography (ICE) probe was advanced to the right atrium via the left femoral venous approach throughout the retrieval procedure to observe TPS movement and to detect any damage to the tricuspid valve or pericardial effusion.

**Figure 1:**
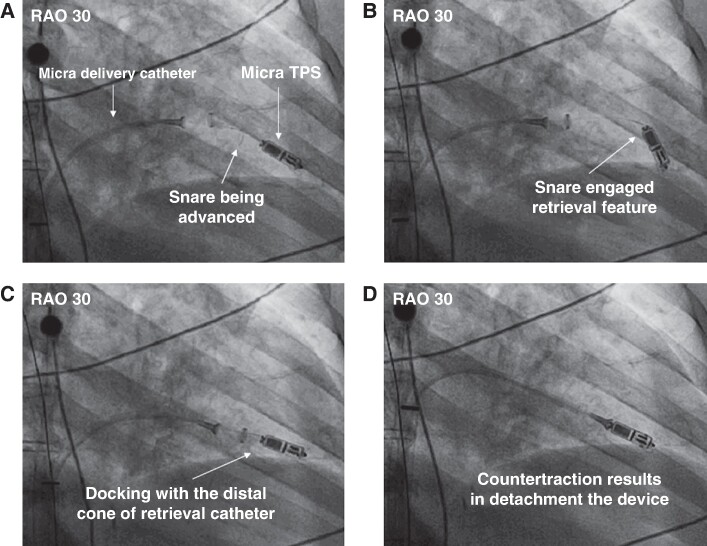
Fluoroscopic views of leadless pacemaker retrieval. (*A*) With the advanced snare catheter, the distal cone of the retrieval catheter (Micra delivery catheter) was deployed around the proximal retrieval feature of the Micra TPS. (*B*) The proximal retrieval feature was snared by closing the loop. (*C*, *D*) The TPS was docked with the cup of the retrieval catheter, and then the constant contra traction resulted in the release of the tines from the RV myocardium, allowing withdrawal of the TPS into the distal cone. RAO, right anterior oblique; TPS, transcatheter pacing system.

After RV contrast injection, a TPS delivery catheter with a single-loop 7 mm snare wire (Amplatz Goose Neck Microsnare Kit, ev3 Inc., Plymouth, MN, USA) was inserted through the 23 Fr sheath. This ‘retrieval system’ was advanced under fluoroscopy and ICE to the junction between the inferior vena cava and the right atrium. The system’s distal cone was manoeuvred into the RV to the proximal portion of the device. The snare was advanced and deployed to grab the TPS ‘retrieval feature’. After confirming coaxial alignment between the snare and the retrieval feature with multi-plane fluoroscopy, the snare was closed and locked around the retrieval feature. The snare loop was then tightened firmly to hold the device, and tension with counter-traction force was applied from the distal cone, to release the tines from the RV myocardium, thereby allowing the withdrawal of the device into the distal cone (*Figure 1*). The delivery catheter and TPS were then withdrawn into the introducer sheath and retrieved outside the patient (see Supplementary material online, *Videos S1–S3*). After retrieval, a new TPS or conventional pacemaker/biventricular pacing system was implanted according to the patient’s clinical indication.

## Results

Transcatheter retrieval of the TPS was performed in nine consecutive patients (mean age 61 ± 14 years, two females) in our institution between March 2016 and November 2023; patient characteristics are shown in *Table 1*. The original indications for LP implantation were (i) atrial fibrillation with slow ventricular rate in four patients, (ii) infrequent cardiac pauses leading syncope in two patients, (iii) repeated device infection of a conventional transvenous pacing system in one patient, (iv) planned surgical tricuspid valve repair in one patient with existing conventional pacing system, and (v) sick sinus syndrome in one patient. All the TPS implantation positions were RV apical-septal segments.

**Table 1:** Patient characteristics

	Age (years)	Gender	BMI	Hypertension	Diabetes	Renal dysfunction	Anticoagulation	Organic heart disease	LVEF (%)	TPS dwell time (years)	Fluoroscopy time (min)	Original indication of TPS implantation	Retrieval indication	Retrieval success	Reimplant device
1	54	M	27	No	No	Yes	Warfarin	Valvular heart disease	40	2.1	18	Planned valve surgery (AVR + TAP)	Planned valve surgery (MAP + epicardial pacing)	Success	DDD
2	77	M	29	Yes	Yes	Yes	DOAC	Ischemic heart disease	60	7.3	5	AF bradycardia	Battery depletion	Success	Micra TPS
3	66	M	35	Yes	Yes	Yes	Warfarin	NA	55	1.1	19	AF bradycardia	Pacemaker syndrome	Success	CRT
4	68	M	37	Yes	No	No	Warfarin	NA	60	0.4	22	AF bradycardia	High pacing threshold	Success	VVI
5	47	M	31	Yes	No	No	NA	NA	55	0.8	32	Syncope due to pause	Pacemaker syndrome	Failure	DDD
6	41	M	24	No	No	No	DOAC	NA	65	2	18	Conventional device infection (DDD for complete AVB)	Planned valve surgery (TAV) and upgrade to DDD	Success	DDD
7	79	F	26	Yes	Yes	Yes	DOAC	NA	60	3.4	15	SSS	Battery depletion	Success	Micra TPS
8	45	M	22	Yes	Yes	No	NA	NA	60	2.2	17	Syncope due to pause	Pacemaker syndrome	Success	ICD
9	72	F	40	Yes	Yes	No	DOAC	NA	55	9	16	AF bradycardia	Battery depletion	Success	Micra TPS
Mean	61 ± 14									3.1 ± 2.8	17.6				

AF, atrial fibrillation; AVB, atrioventricular block; AVR, aortic valve replacement; BMI, body mass index; CRT, cardiac resynchronization therapy; DDD, dual-chamber pacemaker; ICD, intracardiac defibrillator; LVEF, left ventricular ejection fraction; MAP, mitral annuloplasty; SSS, sick sinus syndrome; TAP, tricuspid annuloplasty; TPS, transcatheter pacing system; VVI, single-chamber ventricular pacemaker.

The mean device dwell time in the RV from initial implantation to retrieval attempt was 3.1 ± 2.8 years (range 0.4–9.0 years); in five patients, the TPS retrieval attempt occurred more than 1 year after implantation. The indications for device retrieval were (i) pacemaker syndrome in three patients, (ii) battery depletion in three patients, (iii) planned consecutive tricuspid valve surgery in two patients, and (iv) high pacing threshold in one patient who was dependent on ventricular pacing.

Successful retrieval, defined as complete removal of the LP, was achieved in eight of nine patients (88.9%). The total procedural time was 89 ± 16 min, and the fluoroscopy time was 18.0 ± 6.6 min. In all eight successful retrieval cases, the proximal retrieval feature was snared and held tightly with the delivery catheter. After careful introduction of the distal cone of the delivery catheter over the LP capsule to cover the full body of the implanted TPS, continuous contraction allowed the tines to be released from the endocardial tissue, and the TPS was removed into the distal cone. We also carefully inspected all retrieved devices to observe cardiac tissue remnants. The devices were clear without adherent encapsulations or endocardial tissue components, but only with blood coagulum.

**Figure 2:**
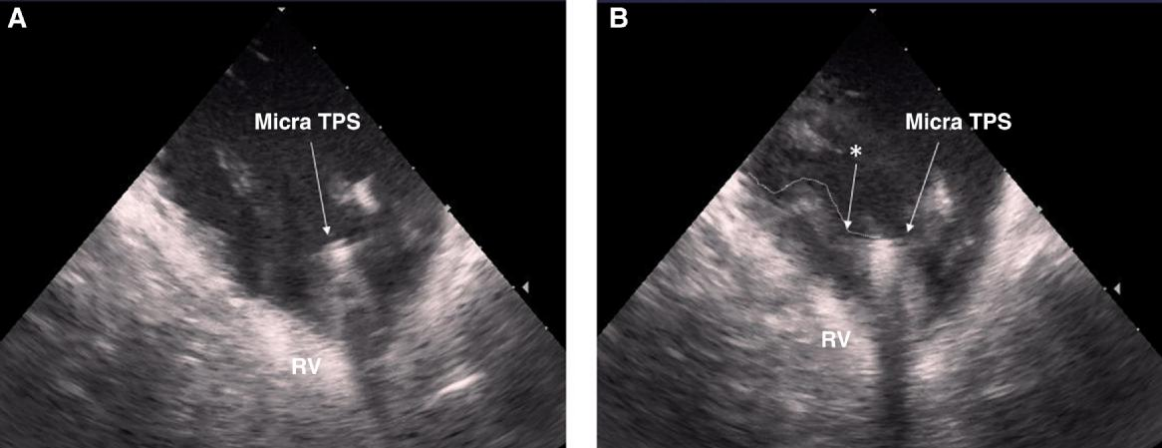
Intracardiac echocardiography. (*A*) Intracardiac echocardiography in a case of successful retrieval of the Micra TPS. The proximal retrieval feature is visible, and the TPS was not covered by surrounding tissue. (*B*) Intracardiac echocardiography in a case of unsuccessful retrieval. Surrounding tissue was severely adherent to the TPS, which was partially embedded within the cardiac endocardium (*denoted by the asterisk). RV, right ventricle; TPS, transcatheter pacing system.

The device retrieval procedure failed in one patient with pacemaker syndrome, 288 days after implantation; the total procedure time of this patient was over 2 h (procedure time: 147 min, and 32 min of fluoroscopy time). In this patient, the 7 mm snare wire was successfully positioned over the rigid proximal retrieval feature knob of the TPS with tight fixation, but we were unable to advance the distal cone over the body and release the pacing capsule from endocardial tissue in the apical part of the septum. As aggressive traction without optimal counter-traction may damage or tear cardiac tissue leading to serious complications such as RV rupture, the retrieval attempt was abandoned without applying further force. Intra-procedural ICE revealed that the TPS body was partially embedded in the endocardial tissue (*Figure 2*). The TPS was deactivated and left abandoned, and a conventional dual-chamber pacemaker was implanted.

Three patients received a new TPS device immediately after retrieval. The previously reported international registry revealed that fibrosis tissue after transvenous lead extraction may affect electrical parameters on LP implantation. In all cases, the new TPS was implanted slightly above the initial location of the retrieved TPS to avoid any fibrous tissue remnant around the old device. All three TPS replacements were successful with normal electrical parameters; the average pacing threshold was 0.73 ± 0.28 V, the average sensing threshold was 11.9 ± 4.6 mV, and the average impedance was 660 ± 79 Ω. One patient with adult congenital heart disease after a surgical operation in his childhood who originally received implantation TPS for planned aortic valve replacement and tricuspid annuloplasty underwent mitral annuloplasty immediately after TPS retrieval. The patient received epicardial leads to upgrade to a dual-chamber (DDD) pacemaker (Patient No. 1, *Table 1*). No procedure-related adverse events were observed in the study.

## Discussion

To the best of our knowledge, this is the largest series demonstrating the safety and feasibility of TPS retrieval with a mid to long RV dwell period using the original TPS implantation tools and a snare catheter. The primary findings of the present study are as follows: (i) retrieval of this tine-designed leadless pacemaker was safe using standard endovascular catheters designed originally for its implantation, and (ii) same-day implantation of a new LP following retrieval of the old system could be a reasonable strategy. As the TPS delivery catheter is provided by the manufacturer as a part of the implantation kit including LP, one of the advantages of the same-day implantation is the straightforward ‘implant-and-retrieve’ procedure. This approach uses the same delivery catheter for both new TPS implantation and the retrieval of the old TPS. In our study, we obtained new TPS delivery catheters and sheaths and utilized detached new LPs for experimental *in vivo* studies. Although the manufacturer recommends abandoning the TPS at end of life, device retrieval may be beneficial and preferred in some scenarios.^13^

To date, there are no detailed studies on the safety and feasibility of the retrieval and reimplantation of TPS devices. In the present study, TPS retrieval was performed based on the patients’ decision only after informed consent with the options of the device retrieval or abandonment as recommended by the manufacturer. Moreover, once endocardial device retrieval failed, new device implantation with either a LP or conventional device was planned. Multiple LP devices could cause device interference or complicate new LP implantation due to the lack of free space. Young patients who require ventricular pacing may reap greater benefit with LP retrieval as the device battery life is expected around 16–17 years.^14,15^

Santobuono *et al*.^16^ reported TPS retrieval 19 months after implantation due to an internal short circuit and sudden battery depletion as they had to remove the device for safety reasons. According to a careful review of the literature, there are not more than 10 case reports of TPS retrieval performed at the late phase after implantation: later than 4 weeks and up to 9 years after implantation.^17–19^ Most of the procedures utilized Agilis NxT deflectable sheath, the Nanostim retrieval catheter (Abbott Inc., St Paul, MN, USA), or a combination of the TPS delivery catheter for implantation and a snare catheter via the central lumen—as employed herein (*Table 2*). The TPS delivery catheter facilitates device capture with a snare as the catheter size and the sheath curve fit the same as when we implant the device. Another unique option reported by Callahan and Wilkoff.^20^ was successful TPS retrieval 5 years after implantation using the retrieval tool for the helix-designed leadless pacemaker. When it comes to the outer sheath sizing, the helix-designed LP retrieval tool has a smaller outer diameter compared with the TPS delivery sheath. The odd sizing might be a limitation for attempts at an older LP retrieval because the retrieval tool targets only the dedicated LP device. In some cases, a combination of a small deflectable sheath and multiple snares could be utilized, but only a few limited size options are currently available to provide efficient counter-traction when attempting to advance the sheath sleeve against the endocardial tissue over the old TPS capsule.

**Table 2:** Recently reported TPS retrievals

Study	Publish	Number	Age, Y	Implant duration	Retrieval success	Retrieval indication	Reimplant device
Minami *et al*.^8^	2020	1	79	1215 days	100%	Battery depletion	TPS
Morita *et al*.^9^	2023	1	78	5 days	100%	Infection	VVI
Patel *et al*.^10^	2023	1	66	49 days	100%	Infection	N/A
Nozoe *et al*.^11^	2018	1	86	8 weeks	100%	Infection	N/A
Fichtner *et al*.^12^	2019	1	83	1 day	100%	Dislodgement	VVI
Grubman *et al*.^13^	2017	5	43–67	5–406 days	60%	N/A	TPS
Santobuono *et al*.^16^	2023	1	85	19 months	100%	Battery depletion	MICRA AV
Karim *et al*.^17^	2016	1	61	21 days	100%	High thresholds	TPS
Curnis *et al*.^18^	2019	1	41	29 months	100%	Battery depletion	TPS
De Filippo *et al*.^19^	2021	1	38	44 months	100%	Battery depletion	TPS
Callahan and Wilkoff^20^	2023	1	38	>5 years	100%	Upgrade to CRT	CRT
Kiani *et al*.^26^	2019	1	78	4 years	100%	Pacemaker syndrome	CRT-D
Chmielewska-Michalak *et al*.^27^	2024	1	76	70 months	100%	Battery depletion	TPS

AF, atrial fibrillation; AV, atrioventricular; CRT-D, cardiac resynchronization therapy defibrillator; TPS, transcatheter pacing system; VVI, single-chamber ventricular pacemaker.

In our study, all the patients had the TPS implanted for at least 134 days and the longest RV dwell time was 9.0 years post-implantation; all patients received a new pacing device, either a new TPS or conventional transvenous pacing system. For most patients, an LP was preferable after retrieval since the first implantation was mainly to prevent device infection and lead/pocket-related complications. We should also consider a device upgrade to a DDD pacemaker, biventricular pacing system, or intracardiac defibrillator (ICD) according to underlying cardiac disease and cardiac function after LP implantation. Five patients in our study underwent upgrade implantation after trials of TPS retrieval considering the progression of reduced ejection fraction, detection of ventricular tachyarrhythmias, or advantages of atrial pacing. With successful TPS retrievals, device selection for the upgrade will be available without limitation, especially since ICD requires good electrical parameters for adequate ventricular tachycardia and ventricular fibrilation detection to avoid fibrous tissue after LP retrieval.^21^ In our study, the pacing/sensing electric parameters were within normal range after the TPS retrievals no matter which pacing device was chosen.

Retrieval was unsuccessful in only one patient even though the proximal retrieval feature of the TPS was snared and fixed to the catheter. The reason for failure to advance the sheath over the TPS to detach the device body from the surrounding endocardial tissue was the incapability of counter-traction due to the device encapsulation. According to past reports, adherent tissue surrounding an implanted TPS device is rare, but a case report of an autopsy with a TPS showed device encapsulation.^22^ Another report noted that a TPS device was covered with fibrous tissue at autopsy less than 1 year after implantation.^23^ Thus, the device encapsulation by fibrous tissue all around the TPS might be associated with challenging retrieval. Intracardiac echocardiography imaging seems to be helpful not only as a navigation modality for retrieval manipulation and the early detection tool of pericardial effusion but also for the assessment of procedural difficulty.

**Figure 3:**
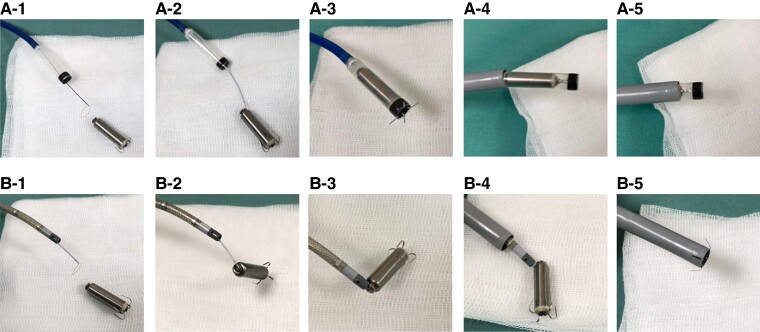
Two approaches for retrieval of Micra TPS retrieval. (*A*) Micra delivery catheter plus snare method and (*B*) steerable sheath plus snare method. (*A-1*) A single-loop 7 mm snare wire is inserted through the central lumen of the Micra delivery catheter introduced through Micra sheath. The system’s distal cone is positioned at the proximal aspect of the device. After the snare advancement, the catheter is deployed around the proximal retrieval feature of the TPS. (*A-2*) The snare is engaged and locked around the retrieval feature of the TPS. (*A-3*) Tension on the snare along with counter traction from the distal cone results in release of the tines from the myocardium, and the system is fully retrieved into the distal cone. (*A-4*, *A-5*) The delivery catheter and TPS are withdrawn into the introducer sheath and removed from the body. (*B-1*) A snare is inserted through a steerable sheath, which can accommodate a snare catheter up to 20 mm. (*B-2*) Engaging the snare to the proximal retrieval feature. (*B-3*) Traction of the snare catheter leads to TPS detachment from the myocardium. (*B-4*, *B-5*) Pulling back the steerable sheath and TPS into the outer sheath.

**Figure 4:**
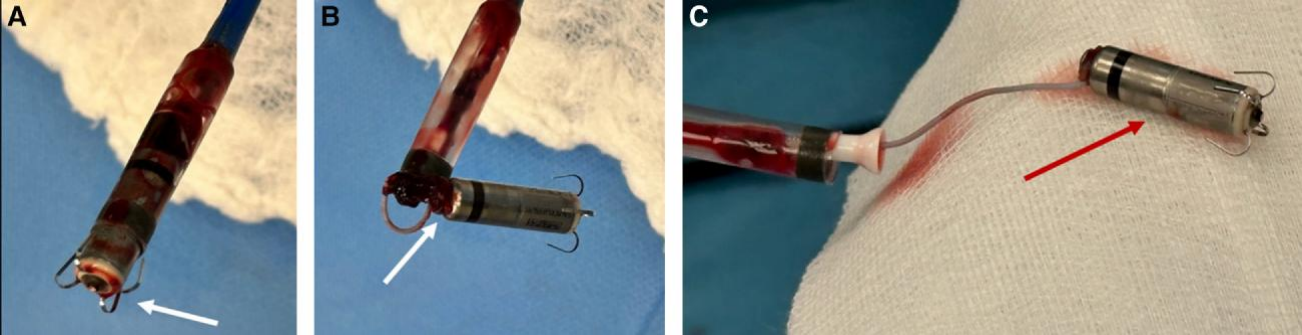
Macroscopic view of retrieved Micra TPS, 9 years after implantation using Micra delivery catheter with 7 mm snare catheter. (*A*) Tines and distal part of TPS are clean (arrow), without any tissue remnants. (*B*) Proximal part of TPS with snare on docking button, the arrow indicates minimal tissue localized around the button. (*C*) On the device capsule, the arrow (out of catheter sleeve) shows no adherent tissue.

When the helix-designed retrieval kit is not available, two main approaches to grab the proximal retrieval feature can be considered (*Figure 3A* and *B*).^24^ Both approaches require femoral venous access through a 23-French TPS introducer sheath but diverge regarding the specific tool employed to engage the TPS during the next step. The snare is advanced through either (i) the integrated protectable sleeve of the TPS delivery catheter or (ii) an 8.5-French steerable sheath.^8,25,26^ In using a steerable sheath, a short sheath (11–16 Fr) is first inserted into the introducer sheath to prevent bleeding from the valve of the TPS introducer sheath, and a steerable sheath (for example, 8.5 Fr, Agilis NXT, Abbott Laboratories, Abbott Park, IL, USA) is inserted and advanced into the RV.^27^ The differences between the two approaches are the snare size variety and the counter-traction force to detach the TPS from the endocardial tissue. That is, it is easier to snare the retrieval feature using a steerable sheath because this sheath accepts a 20 mm diameter loop snare or a tri-loop snare. However, to withdraw, the TPS is occasionally challenging due to its small diameter. In contrast, the TPS delivery catheter can accommodate only a 7 mm snare catheter through the central lumen, but the 7 mm snare is feasible to engage the proximal retrieval feature of the TPS (*Figure 4*; Supplementary material online, *Video S4*). The advantage of using the TPS delivery catheter is to provide optimal counter-traction to remove the surrounding endocardial tissue around the device body using the distal cone.^28^ Another advantage is the simple setup when old and new TPS exchange is required. Although we performed TPS retrieval first and new TPS implantation followed so as not to affect the new TPS stability in all three TPS exchanges, the TPS delivery catheter can be re-utilized to retrieve an old TPS with a 7 mm snare catheter after new TPS implantation next to the old device as long as all retrieval manipulations are away from the newly implanted device.^16^ In any event, contra-traction and counter-traction with the selected retrieval tools play a key role in retrieval, unlike the helix-fixation LP retrieval with a dedicated retrieval catheter kit enabling efficient counterclockwise rotation to unscrew the device from the myocardium in the RV. The conditions for a successful retrieval procedure need to be clarified because both helix and tine fixation LP successful retrievals were reported up to 9 years after implantation.^29^

### Limitations

This study has several limitations. First, it is a non-randomized observational single-centre experience with a small sample size. Second, the learning curve for the procedure may have influenced the procedure time, fluoroscopy time, and retrieval success rate. Third, the combination of the TPS delivery catheter and a snare catheter was utilized for all the attempted cases, and no other alternative options were evaluated. However, the indication for device retrieval was limited, and the patient’s desire and decision were primarily followed. Experienced operators performed all retrieval procedures in this study to respect safety. A large number of retrieval attempts should be investigated to understand when we should decide to retrieve or not to retrieve the device or who is the high-risk patient.

## Conclusions

Retrieval of the TPS device with mid- to long-term RV dwell period after implantation was safely performed, which may indicate the potential benefits for patients and feasibility of same-day LP replacement.

## Supplementary Material

euae256_Supplementary_Data

## Data Availability

The data underlying this article will be available on reasonable request to the corresponding author.
